# Aditus ad Antrum on Normal Temporal Bone CT Scan

**DOI:** 10.1055/a-2825-9870

**Published:** 2026-08-14

**Authors:** Son Bao Nguyen, Chat Ngoc Pham, Vinh Thanh Nguyen, Minh Thieu Quach

**Affiliations:** 1 University of Medicine and Pharmacy, Ho Chi Minh City, Vietnam; 2 Department of Otolaryngology, University of Medicine and Pharmacy, Ho Chi Minh City, Vietnam; 3 Ear Nose Throat Hospital, Ho Chi Minh City, Vietnam

**Keywords:** temporal bone, mastoid, middle ear ventilation

## Abstract

**Introduction:**

The aditus ad antrum is a structure located on the posterior wall of the tympanic cavity, connecting the epitympanum and mastoid antrum, playing a role in ventilating the mastoid antrum and air cells. However, there is limited in-depth research on this structure.

**Objective:**

To study the anatomy and morphology of the aditus ad antrum and evaluate its correlation with mastoid pneumatization using 3D MPR-reconstructed CT scans.

**Methods:**

This cross-sectional descriptive study was conducted using 101 normal temporal bone CT scans (190 ears) from individuals aged 16 years or older in Vietnam. The aditus was examined in three planes (axial, coronal, and sagittal) using 3D MPR reconstruction.

**Results:**

The aditus ad antrum morphology was classified into four groups, with group 2 being the most prevalent (48.42%). There was no correlation between aditus morphology and gender or ear side. Additionally, no relationship was found between mastoid pneumatization and aditus morphology. With increasing mastoid pneumatization, aditus ad antrum dimensions change. In individuals with two normal ears ( *N* = 89), bilateral symmetry in aditus morphology was observed in 45% of cases. No significant differences were found in aditus dimensions between ears. The mean transverse diameter of the aditus in males was significantly larger than in females ( *p* < 0.05). The anterior-posterior diameter of the right ear was significantly greater than the left in the same individual ( *p* < 0.05).

**Conclusion:**

The aditus ad antrum may differ between the left and right ears in normal individuals and is not associated with mastoid pneumatization.

## Introduction

The aditus ad antrum (AAA) is a middle ear structure located in the upper third of the posterior wall of the tympanic cavity, forming a connection between the epitympanum and the mastoid antrum. The eustachian tube ventilates the tympanic cavity (including the hypotympanum, mesotympanum, and epitympanum) and the mastoid air cells via the AAA. 1 The patency of the AAA ensures adequate ventilation of the mastoid antrum and air cells. 2 Obstruction of the AAA can impair mastoid pneumatization, potentially contributing to the failure of tympanoplasty. 2 3 4 Therefore, preoperative evaluation of AAA patency is essential for guiding surgical strategies (tympanoplasty, mastoidectomy, or a combination) in various middle ear pathologies.

The AAA is an important landmark in surgery. Traditionally, to access the epitympanum posteriorly (posterior epitympanotomy), surgeons prefer to first open the mastoid antrum followed by the AAA. In certain situations, direct opening of the AAA via a postauricular or transcanal approach is necessary to reach the pathology. Recently, cochlear implantation via the AAA (trans-aditus approach) has been considered an advantageous alternative, particularly when the traditional facial recess approach is not feasible. 5

There have been several studies worldwide on the dimensions and obstruction of the AAA across different populations. 6 7 8 9 However, most researchers have considered the AAA as a two-dimensional structure and have employed various measurement techniques. Although the AAA has long been discussed in the medical literature, its boundaries, shape, and relationship with mastoid pneumatization have not been clearly described.

In this study, we conducted an anatomical and morphological assessment of the AAA using CT scans with 3D multiplanar reconstruction (3D MPR). We conceptualized the AAA as a three-dimensional cylindrical structure with defined anatomical landmarks. Along with measuring the dimensions of the AAA, we classified its variations and examined the association and correlation of this structure with the extent of mastoid pneumatization. This study contributes to the anatomical understanding and theoretical framework of the shared developmental mechanisms between mastoid pneumatization and the AAA. Additionally, the findings assist surgeons in preoperative AAA evaluation, providing a general understanding of its structure to facilitate surgical planning and to help avoid complications such as dural exposure or injury, as well as unintended damage to the superior wall of the external auditory canal.

## Methods

This cross-sectional descriptive study was conducted using temporal bone CT scans of participants obtained from September 2023 to July 2024. Inclusion criteria included temporal bone CT scans from individuals aged 16 years or older, confirmed by radiologists to have a normal ear on the side being examined. The primary imaging plane was axial, with reconstructed coronal and sagittal planes, ensuring that all anatomical landmarks were clearly visible. Exclusion criteria included middle ear or mastoid pathologies, congenital temporal bone anomalies, temporal bone fractures, prior middle ear surgery on the examined side, and age under 16.

Imaging data were acquired using a Siemens Emotion 64-slice CT scanner with a slice thickness of 0.6 mm. Image analysis was performed using OsiriX MD software (version 14.0.1). All measurements were performed by a radiologist specializing in ear, nose, and throat pathologies.

We defined specific anatomical landmarks to facilitate the measurement and classification of the AAA. The lateral boundary of the AAA is the continuous bony wall of the lateral epitympanum extending posteriorly. The medial boundary is the bony wall separating the middle ear from the inner ear. The superior boundary is the mastoid tegmen. The anterior boundary is marked by the incudal buttress. The posterior boundary is defined by drawing an imaginary circle encompassing the margins of the mastoid antrum, with the anterior tangent line of this circle serving as the posterior boundary.

**Fig. 1:**
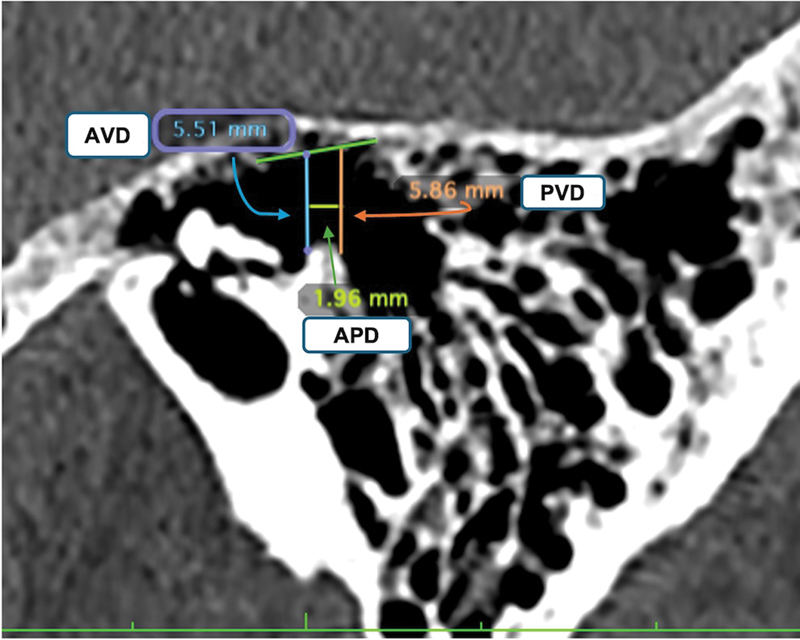
Illustration of measurements for the anterior vertical dimension (AVD), posterior vertical dimension (PVD), and anteroposterior dimension (APD) of the aditus ad antrum.

**Fig. 2:**
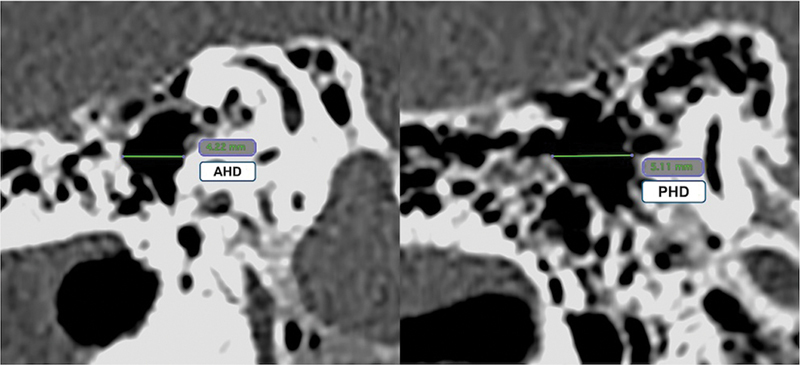
Illustration of measurements for the anterior horizontal dimension (A) and posterior horizontal dimension (B) of the aditus ad antrum.

On the selected axial plane, the coordinate axis was rotated along the axis of the incudomalleolar joint. On the corresponding sagittal plane, a point was selected on the first slice intersecting the incudal buttress. The superior boundary of the AAA was marked with a continuous line passing through the mastoid air cells at the tegmen. The anterior vertical dimension (AVD) was measured on the sagittal slice, while the anterior horizontal dimension (AHD) was measured at its largest point on the corresponding coronal slice. The posterior boundary of the AAA is defined by a tangent line to an imaginary circle encompassing the limits of the mastoid antrum. The posterior vertical and horizontal dimensions (PVD and PHD) are then measured accordingly. The anteroposterior dimension (APD) was determined by connecting the anterior and posterior boundaries of the AAA. Fig. 1 illustrates authors' measuring methods of anterior vertical dimension, posterior vertical dimension and anteroposterior dimension of the AAA. Fig. 2 illustrates the measurements of anterior horizontal dimension and posterior horizontal dimension of the AAA.

**Fig. 3:**
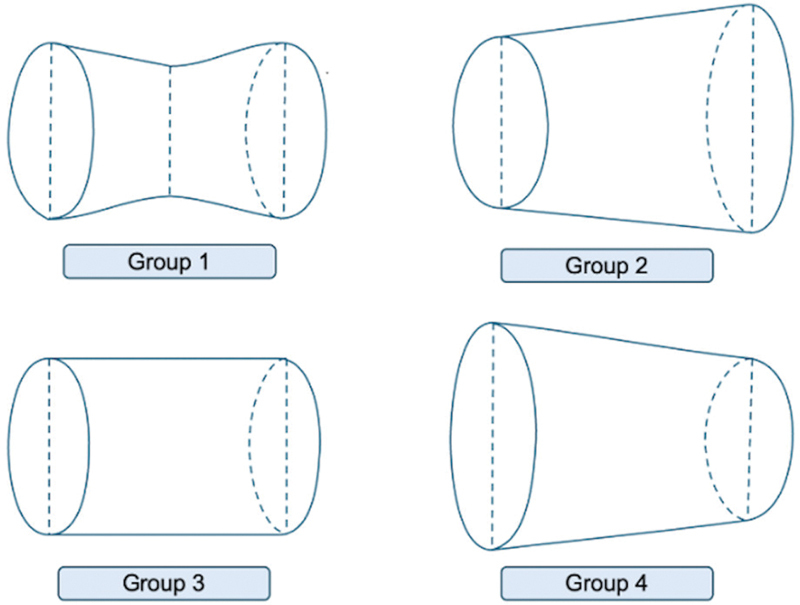
Classification of aditus ad antrum shapes.

**Fig. 4:**
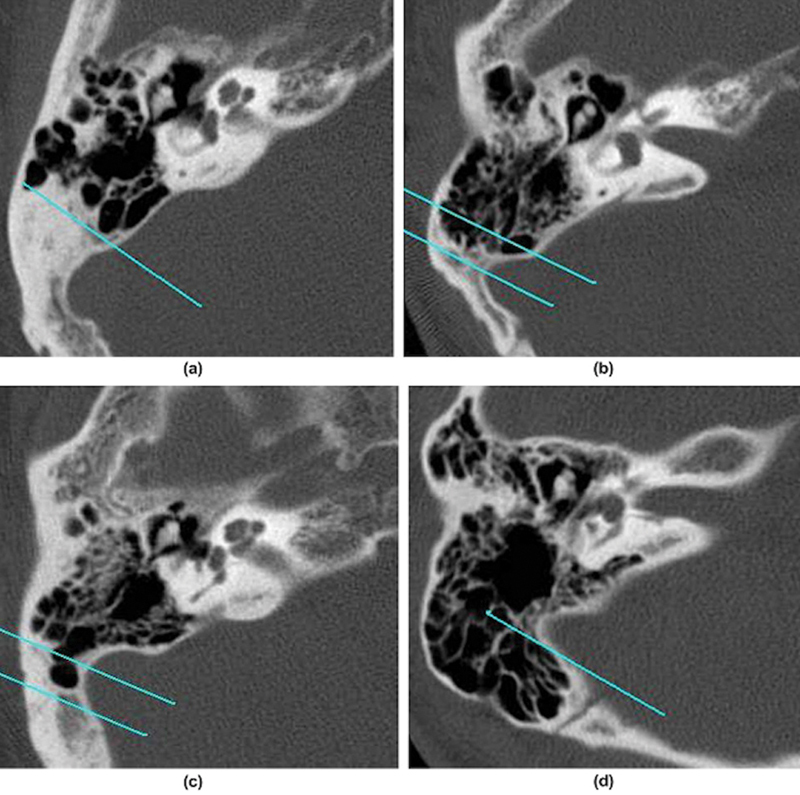
Classification of mastoid pneumatization in relation to the sigmoid sinus (Han S.J., 2007). 10 (A) Hypopneumatization; (B) Moderate pneumatization; (C) Good pneumatization; (D) Hyperpneumatization. The parallel lines angled at 45° in the anterolateral direction were applied for the classification. The lines cross the most anterior point of the sigmoid sinus, the most lateral aspect along the transverse plane of the sigmoid groove and the most posterior point of the sigmoid sinus, respectively.

The AAA was classified into four groups based on the difference between the anterior and posterior dimensions and the presence or absence of a waist-like constriction ( Fig. 3 ). Group 1: Presence of a constriction in the middle of the structure. Group 2: The PVD is larger than the AVD by more than 0.5 mm. Group 3: The difference between the AVD and PVD is less than 0.5 mm. Group 4: The AVD is larger than the PVD by more than 0.5 mm. For cases classified into group 1, we measured the middle vertical and middle horizontal dimensions (MVD and MHD) on the sagittal and coronal planes, respectively. The degree of mastoid pneumatization was assessed using the Han S.J. classification ( Fig. 4 ), 10 which evaluates the extent of pneumatization in relation to the sigmoid sinus. The correlations between AAA dimensions and the degree of mastoid pneumatization were then analyzed.

Data were analyzed using SPSS Statistics version 27. An independent *t* -test was used to compare the means between two groups, while a paired *t* -test was applied for measurements within the same individual. Chi-square (χ ^2^ ) and Fisher's exact tests were use to analyze the association between the degree of mastoid pneumatization, ear side, sex, and the AAA shape. Spearman's rank correlation test was employed to assess the correlation between the dimensions of the AAA and the degree of mastoid pneumatization. A p-value of < 0.05 was considered statistically significant.

## Results

This study included 101 CT scans that met the inclusion criteria, corresponding to 190 ears. Among these, 89 scans showed bilateral normality, while the remaining 12 ears were selected from scans with unilateral pathology. Of the 190 ears examined, left and right ears were equally represented, each accounting for 50%. The study sample consisted of 50 males (49.5%) and 51 females (50.5%). Participant ages ranged from 16 to 75 years, with a mean age of 45.30 ± 14.98 years.

### Dimensions and Classification of the Aditus Ad Antrum

The detailed measurements of the AAA dimensions are presented in Table 1 . Only 7 cases classified into group 1 were assessed for MVD and MHD. When analyzed by ear side, the differences in AAA dimensions were not statistically significant. However, by sex, the AHD and PHD were significantly larger in males than in females ( *p* = 0.004 and *p* < 0.001, respectively). Within the same individual ( *N* = 89), the APD of the right ear was significantly larger than that of the left ( *p* = 0.032).

**Table 1:** Dimensions of the aditus ad antrum (mm)

Dimensions (mm)	Number of ears	Average (mm)	Range (mm)	95% CI (mm)
**Anterior vertical**	190	6.29 ± 1.32	2.98 - 10.1	6.10 - 6.48
**Posterior vertical**	190	6.54 ± 1.48	2.47 - 9.91	6.33 - 6.75
**Anterior horizontal**	190	5.70 ± 1.18	3.51 - 9.11	5.53 - 5.87
**Posterior horizontal**	190	5.72 ± 1.31	3.18 - 13.5	5.53 - 5.90
**Anteroposterior**	190	1.56 ± 0.58	0.51 - 3.25	1.47 - 1.64
**Middle vertical**	7	4.15 ± 0.43	3.48 - 4.70	−
**Middle horizontal**	7	5.32 ± 1.22	3.27 - 6.87	−

Abbreviation: 95% CI, 95% confidence interval.

**Fig. 5:**
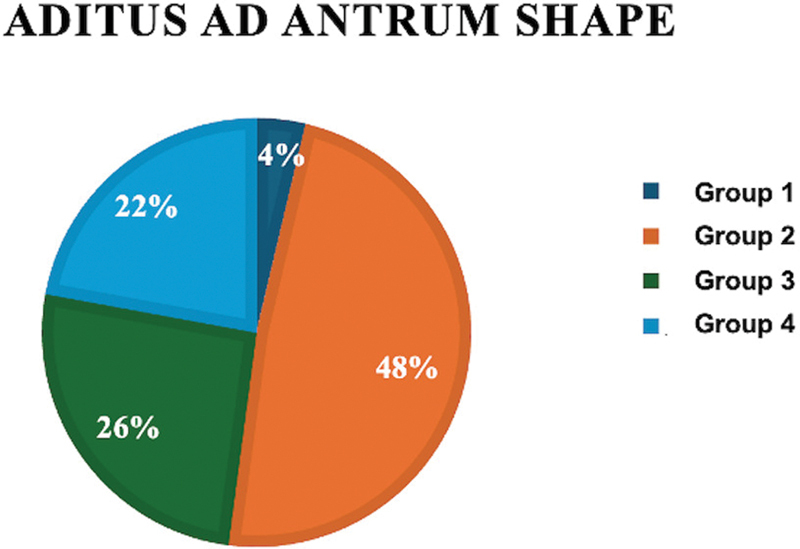
Distribution of aditus ad antrum groups.

Regarding the shape of the AAA, Group 2 was the most prevalent, accounting for 92 cases (48.42%), followed by group 3 (49 cases, 25.79%), group 4 (42 cases, 22.11%), and group 1 (7 cases, 3.68%) ( Fig. 5 ). No association was found between the AAA shape and sex or ear side.

### Mastoid Pneumatization and Aditus Ad Antrum

Among the 190 ears examined, hyperpneumatization was the most prevalent finding (71.05%), followed by good pneumatization (20.00%). Hypopneumatization was observed in only 1 case (0.53%). No association was found between the degree of mastoid pneumatization and gender or the side of the ear examined. Among the 89 scans with bilateral normality, 72% showed symmetrical mastoid pneumatization. In contrast, AAA shapes were classified identically on both sides in only 45% of cases. There was no association between the shape of the AAA and gender or ear side. Additionally, no association was found between the degree of mastoid pneumatization and the shape of the AAA, regardless of gender or ear side examined.

**Fig. 6:**
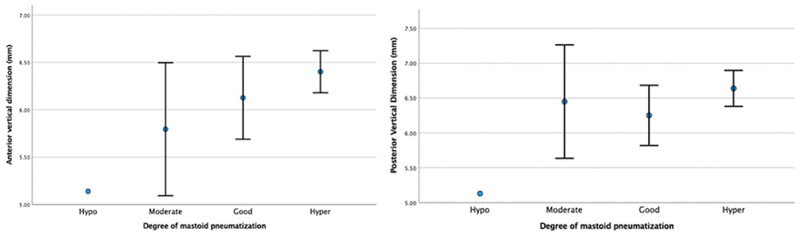
Anterior and posterior vertical dimensions by degree of mastoid pneumatization.

**Fig. 7:**
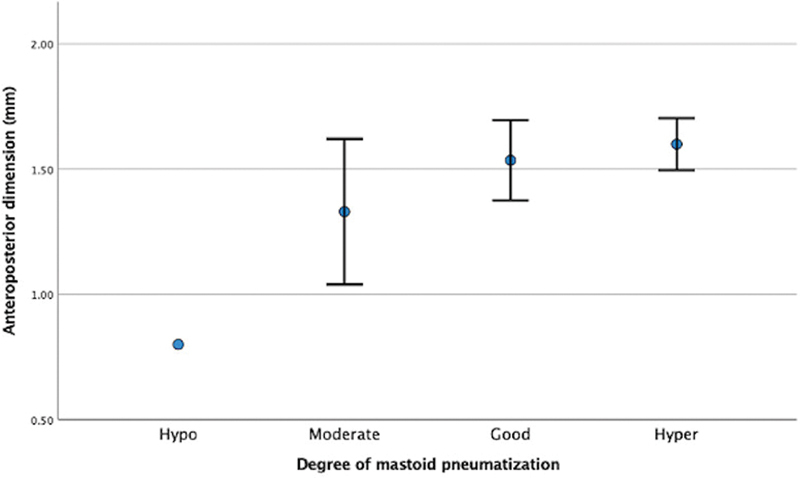
Anteroposterior dimension by degree of mastoid pneumatization.

**Fig. 8:**
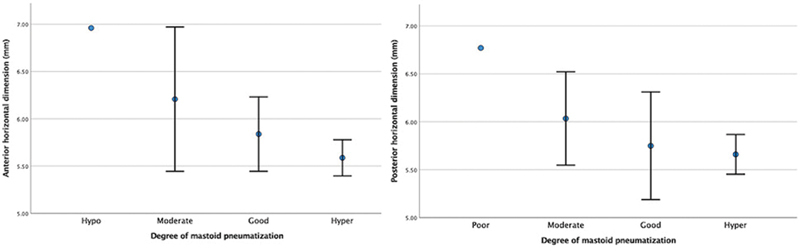
Anterior and posterior horizontal dimensions by degree of mastoid pneumatization.

Analysis of the correlation between AAA dimensions and mastoid pneumatization revealed that as pneumatization increased from hypopneumatization to hyperpneumatization, the AVD, PVD, and APD tended to increase ( Fig. 6 and Fig. 7 ). Among these, the correlation with the AVD was statistically significant (Spearman's rho = 0.153; *p* = 0.035). In contrast, the AHD and PHD showed a decreasing trend. This negative correlation (rho = -0.142 and rho = -0.080) was not statistically significant (p > 0.05) ( Fig. 8 ).

## Discussion

This is the first study to provide a detailed assessment of the dimensions and classification of the AAA using 3D MPR-reconstructed temporal bone CT scans. The findings contribute to the development of an anatomical model of the AAA, serving as a reliable reference for future research. Additionally, the results also aid in preoperative planning for relevant surgical procedures.

Regarding the dimensions of the AAA, our study examined various measurements across multiple planes. Notably, the mean AVD and PVD were 6.29 ± 1.32 mm (95% CI: 6.10-6.48 mm) and 6.54 ± 1.48 mm (95% CI: 6.33-6.75 mm), respectively. The average AHD and PHD were 5.70 ± 1.18 mm (95% CI: 5.53-5.87 mm) and 5.72 ± 1.31 mm (95% CI: 5.53-5.90 mm), respectively. Previous studies have primarily considered the AAA as a 2D structure, with vertical and horizontal dimensions often assessed using different methodologies. Our results were significantly larger than those of Long et al. (2012), 7 likely due to differences in sampling criteria, as their study included participants as young as 1 year old, with one-third under 6 years of age – an age range during which the mastoid bone is still developing. Bahgat et al. (2014) 2 examined pathological samples, using square hooks of varying sizes for measurements after widening the mastoid antrum. In addition to disease-related changes, the use of drills alters the native AAA structure, which likely accounts for the differences in results compared with those of our study. The studies by Elif Gündoğdu (2019), 9 Cavaliere (2023), 11 and Fernández-Reyes (2024) 12 measured the horizontal dimension of the AAA at its narrowest point on the axial plane, specifically at the level of the lateral semicircular canal bulge. Fernández-Reyes also assessed the vertical dimension but only drew a straight line connecting the superior wall of the external auditory canal to the tegmen area, without rotating the axis along the incudomalleolar joint. This approach did not fully capture the true dimensions of the AAA, which likely accounts for the smaller measurements reported in these three studies compared with our findings.

Regarding the shape of the AAA, this is the first study worldwide to investigate and classify its variations. According to some sources, the AAA is described as a short, restricted bony canal situated at the posterior prolongation of the attic, typically triangular in shape with dimensions of 4 × 4 × 4 mm. 1 However, the present study reveals that this description does not encompass all cases. In reality, the AAA is a three-dimensional structure with various morphological variations, extending beyond a simple triangular form. No association was found between the degree of mastoid pneumatization and AAA shape. Although group 2 AAA was predominant in both sexes and on both sides, no relationship was observed between AAA shape and the degree of mastoid pneumatization, regardless of sex or ear side. Among the 89 CT scans with bilateral normality, bilateral symmetry in AAA shape was observed in only 45% of cases, compared with 72% for mastoid pneumatization. This suggests that the shape of the AAA may differ between sides and is unrelated to the bilateral symmetry of mastoid pneumatization. In summary, AAA shape does not depend on the degree of mastoid pneumatization, sex, or ear side. This finding suggests that other factors, potentially genetic or environmental, may play a significant role in determining the shape of the AAA.

Based on the present findings, the AVD of the AAA was selected as the base for a classification into small, medium, and large groups. The rationale for this choice is that the AAA entrance (posterior wall of the epitympanum, corresponding to the AVD) represents the narrowest point when accessing the epitympanum posteriorly. Anatomically, the dura mater tends to slope downward from posterior to anterior on the sagittal plane and becomes closer to the superior wall of the external auditory canal as it approaches the epitympanum. 1 Additionally, the AVD followed a normal distribution and showed a statistically significant positive correlation with the degree of mastoid pneumatization. Knowledge of this dimension could influence clinical decisions, such as selecting the appropriate drill size for accessing the epitympanum from the mastoid antrum or informing preoperative planning. This may help reduce the risk of unintended dural exposure, dural injury, or drilling into the superior wall of the external auditory canal.

Dimensions were classified into three categories: group 1 for AVD < 4.2 mm (< 5th percentile), group 2 for AVD between 4.2 and 8.21 mm, and group 3 for AVD > 8.21 mm (> 95th percentile). To better suit clinical practice, an adjusted classification of AAA dimensions is proposed, as shown in Table 2 . These dimensions can be measured on both sagittal and coronal planes once the AAA has been identified.

**Table 2:** Classification of the aditus ad antrum dimensions based on the AVD

	Small	Medium	Large
**Dimension (mm)**	0-4.0	4.0-8.0	> 8.0

A limitation of the present study is the inability to generate a 3D reconstruction of the AAA due to constraints of the available research tools. In reality, the structure of the AAA may be far more complex, requiring studies with 3D reconstruction capabilities for a more precise assessment of its morphology, dimensions, and volume. This necessitates a standardized approach regarding anatomical landmarks and measurement techniques. Additionally, further studies on normal temporal bone specimens from cadaveric dissection, with comparison to CT images with thinner slice thickness, are warranted. This would help validate the accuracy of the results, develop a more detailed classification system, and provide clearer clinical recommendations.

In addition to providing parameters for preoperative evaluation, the results of this study offer baseline reference data on the normal dimensions of the AAA, which may support future research investigating the structural changes of this region in the presence of middle ear diseases. Furthermore, a feasible research direction would be the combination of aditus dimensions with parameters of other middle ear structures or the eustachian tube to predict the risk of middle ear or epitympanic disease, as well as surgical failure. Such in-depth studies may contribute to elucidating the pathogenesis of middle ear disease, in which dysfunction of the natural ventilation pathways has consistently been recognized as an important mechanism.

## Conclusion

The aditus ad antrum is a crucial middle ear structure that helps maintain mastoid aeration through the eustachian tube. In addition to its role in the pathogenesis of middle ear diseases, the AAA also serves as an important surgical landmark. The results showed several differences when comparing bilateral AAA, including variations in dimensions and shape, as well as dimensional changes related to the degree of mastoid pneumatization. These findings provide a strong foundation for further in-depth studies on the AAA, which is one of the key pathways for middle ear aeration. Ultimately, this may lead to more meaningful clinical recommendations.
